# Thioridazine inhibits angiogenesis and tumor growth by targeting the VEGFR-2/PI3K/mTOR pathway in ovarian cancer xenografts

**DOI:** 10.18632/oncotarget.2063

**Published:** 2014-06-06

**Authors:** Mi Sun Park, Seung Myung Dong, Boh-Ram Kim, Seung Hee Seo, Sokbom Kang, Eun-Ju Lee, Seung-Hoon Lee, Seung Bae Rho

**Affiliations:** ^1^ Research Institute, National Cancer Center, 323, Ilsan-ro, Ilsandong-gu, Goyang-si Gyeonggi-do, Republic of Korea; ^2^ Department of Obstetrics and Gynecology, Chung-Ang University School of Medicine/Chung-Ang University Hospital, Seoul, Republic of Korea; ^3^ Department of Life Science, Yong In University, 470, Samga-dong, Cheoin-gu, Yongin-si Gyeonggi-do, Republic of Korea

**Keywords:** thioridazine, anti-tumor effect, anti-angiogenic activity, mTOR signaling, xenograft model

## Abstract

Thioridazine, a member of the phenothiazine family, is a powerful anti-anxiety and anti-psychotic drug. It can also suppress the growth of several types of tumor *in vitro*. In the current study, we evaluated the direct anti-tumor and anti-angiogenic effects of thioridazine *in vivo*. The injection of thioridazine into human ovarian tumor xenografts in nude mice significantly inhibited tumor growth by ~fivefold, and also decreased tumor vascularity. In addition, thioridazine inhibited the phosphorylation of the signaling molecules downstream of phosphatidylinositol-3’-kinase (PI3K), including Akt, phosphoinositide-dependent protein kinase 1 (PDK1), and mammalian target of rapamycin (mTOR), during ovarian tumor progression via vascular endothelial growth factor receptor 2 (VEGFR-2). These results provide convincing evidence that thioridazine regulates endothelial cell function and subsequent angiogenesis by inhibiting VEGFR-2/PI3K/mTOR signal transduction. Collectively, these results strongly suggest that thioridazine might be a novel anti-tumor and anti-angiogenic agent for use in ovarian cancer.

## INTRODUCTION

Thioridazine hydrochloride (10-[2-(1-methyl-2-piperidyl)ethyl]-2-methylthiophenothiazine) is one of several anti-psychotic drugs collectively named phenothiazine. It is commonly used to treat schizophrenia and other psychotic disorders. Thioridazine decreases excitation, agitation, hypermobility, and abnormal conditions associated with excess energy. In individuals with advanced tumors, it is also widely used to treat tumor-associated sweating [1, 2] and depression [3]. Previous studies have shown that thioridazine exerts several biological effects, including reducing the levels of P-glycoprotein [4] and DNA breakdown [5]. Recently, we reported that thioridazine inhibits cell proliferation by inducing G_1_ cell cycle arrest and induces apoptotic cell death. In addition, the anti-proliferative and anti-angiogenic effects of thioridazine on ovarian carcinoma cells might be caused by inhibition of the PI3K/Akt and FAK/ mammalian target of rapamycin (mTOR) pathways *in vitro* [6, 7]. However, the effect of thioridazine on ovarian tumor progression *in vivo* remains unclear.

Angiogenesis is a physiological multi-step process that includes endothelial cell growth and movement. It plays important roles in wound healing and endothelial-cell-mediated degradation of the extracellular matrix, as well as the transition of benign tissue into solid tumors [8, 9]. Vascular endothelial growth factor (VEGF) is a key activator of endothelial cell functions such as new blood vessel formation during development. It also plays a vital role in the proliferation, migration, and invasion of vascular endothelial cells [10]. Growth factors, such as VEGF, integrins, and growth factor receptors (GFRs) stimulate angiogenesis. Specifically, biological signals known as angiogenic growth modulators activate receptors on the surface of endothelial cells in pre-existing vessels [8, 11].

Recent studies have suggested that the inhibition of PI3K might play a vital role in tumor angiogenesis [12-14]. During apoptotic cell death, the apoptosis signal transduction pathway modulated by Akt is activated via PI3K; Akt is a pivotal downstream target of PI3K during angiogenesis. Akt regulates multiple cellular processes including tumor angiogenesis, cell cycle progression, cell growth, cell migration, and cell metabolism [15, 16]. In animal experiments, the siRNA-mediated suppression of Akt effectively downregulated ovarian tumor growth and angiogenesis [12, 14]. Therefore, the PI3K/Akt signaling cascade plays a vital role in tumor angiogenesis. mTOR is also a critical regulator of cell growth and death; it functions by modulating a variety of signal transduction pathways [17, 18].

The current study used an *in vivo* model of human ovarian cancer cell xenografts in nude mice to assess the effects and mechanism of action of thioridazine on tumor growth and angiogenesis.

## RESULTS

### Thioridazine inhibits the growth of 2774 xenografts in nude mice

**Figure 1:**
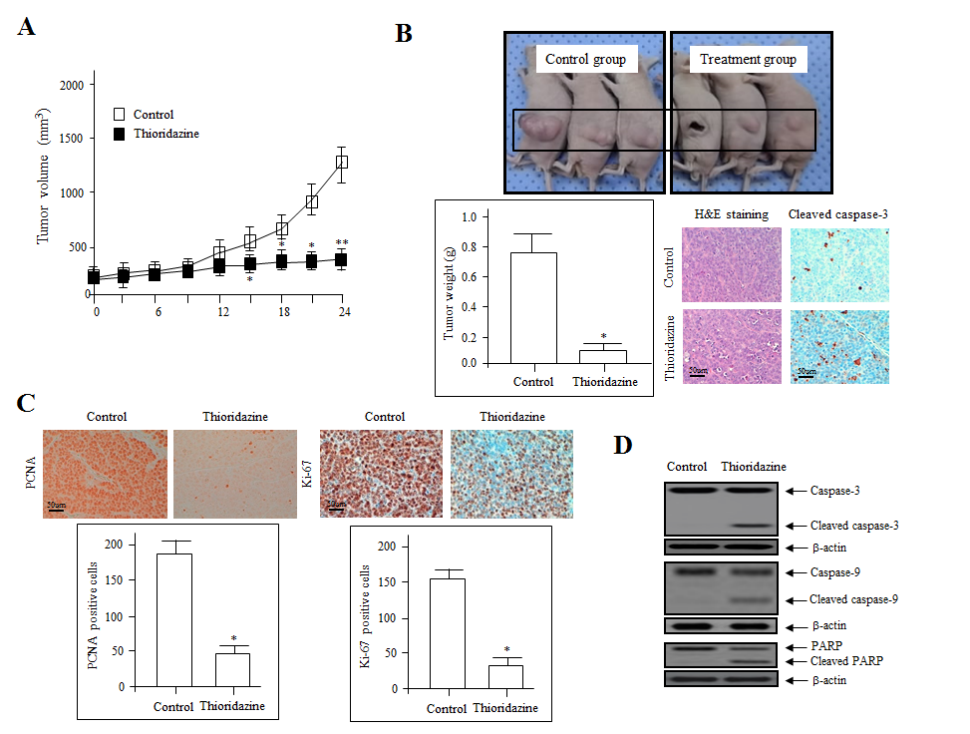
Thioridazine inhibits tumor growth *in vivo* (A) 2774 tumor cells were injected s.c., control (□) and thioridazie (▀) treatments were performed orally every 3 days. Data represent the mean tumor volume of four to five mice. *, *P*<0.05; **, *P*<0.01 compared with the controls. (B) The tumors excised from control and thioridazine-treated mice on day 28 of treatment were photographed (upper panel). Tumor weights are reported as the mean of three mice (*n* = 5 per group) (lower left panel) ± SDs. *, *P*<0.05 vs. control. Sections of thioridazine-treated and control tumors were stained with hematoxylin and eosin (H&E; lower middle panel) and cleaved caspase-3 (lower right panel) to assess cell morphology and apoptosis. Bars = 50 μm. (C) Immunohistochemical staining for the cell proliferation markers PCNA and Ki-67. PCNA and Ki-67 expression was decreased significantly in the thioridazine-treated group compared with the controls. Bar = 50 μm. *, *P*<0.05 vs. control. (D) Immunoblotting for cleaved and total caspase-3, caspase-9, and PARP in 2774 xenografts from nude mice treated with control and thioridazine; β-actin was used as the loading control.

To investigate whether thioridazine exerts direct anti-tumor and anti-angiogenic effects, we evaluated its effects on the growth of ovarian cancer xenograft tumors *in vivo*. Ovarian cancer cells (2774) were injected subcutaneously into nude mice, and were allowed to develop into ovarian tumor xenografts. The volume of tumors in the control group increased significantly after 14 days (mean volume = 100 mm^3^). Beginning on day 14, mice in the treatment group were treated orally with 25 mg/kg thioridazine every 3 days for 4 weeks. Tumor growth in the thioridazine-treated group was suppressed ~fivefold compared with control (Fig. 1A).

Tumors were excised from both groups on day 28 after the final treatment. The volume of thioridazine-treated tumors was 70% less than those from control mice. Treatment resulted in no overt toxicity in organ tissue sections, no significant toxic lesions (data not shown). Hematoxylin and eosin staining of tumor sections from the control group revealed high-grade carcinoma with an irregular cell distribution and mitotic morphology. In contrast, tumors from the thioridazine-treated group had large areas of late-apoptotic or necrotic cells. Cleaved caspase-3 immunohistochemical findings indicated the apoptotic effect by thioridazine (Fig. 1B). Treated and control group tissues were also stained for proliferating cell nuclear antigen (PCNA) and Ki-67, markers of cell proliferation. The expression of PCNA and Ki-67 was significantly lower in the thioridazine treatment group compared with the controls (Fig. 1C).

We next determined whether thioridazine induces apoptotic cell death by evaluating the proteolytic activity of caspase-9 and caspase-3, precursors of major regulators of apoptosis, as well as the inactivation of poly (ADP-ribose) polymerase (PARP), using immunoblotting. As shown in Fig. 1D, in a manner consistent with the morphological characteristics of apoptotic cell death such as fragmented nuclei, increased dosages of thioridazine stimulated cleavage of PARP. We monitored PARP cleavage as a significant indicator, as well as a useful marker for cellular apoptotic cell death outcomes. We also measured the activation of caspase-3, which plays an essential role in the execution of apoptosis [19]. The expression of the cell-death-associated proteins PARP, caspase-9, and caspase-3 was enhanced significantly in thioridazine-treated tumors. These results suggest that thioridazine inhibits tumor growth by inducing apoptosis *in vivo*.

### Effect of thioridazine on the expression of cell cycle-regulatory and apoptosis-associated proteins

**Figure 2:**
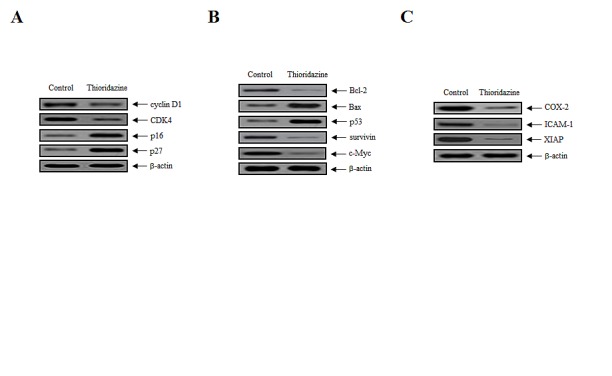
Thioridazine induces apoptosis in ovarian tumor xenografts by downregulating the expression of cell cycle and anti-apoptotic proteins (A) Whole protein extracts were isolated from xenograft tumors, and the expression of cyclin D1, CDK4, p16, and p27 was analyzed by immunoblotting. (B, C) The expression of the anti-apoptotic, oncogenic and anti-proliferative proteins Bcl-2, Bax, p53, survivin, c-Myc, COX-2, ICAM-1, and XIAP was analyzed by immunoblotting; β-actin was used as the loading control.

To clarify the biological mechanism underlying the growth inhibitory effects of thioridazine, the expression of cell-cycle-associated proteins in tumor tissue lysates was assessed. The expression of cyclin D1 and cyclin dependent kinase 4 (CDK4), which are associated with the transition from G_1_ to S phase, was downregulated significantly, whereas the expression of the CDK inhibitors p16 and p27, which interrupt cell cycle procession at the G_1_ or G_2_/M phase, was increased (Fig. 2A). In addition, the expression of Bax and p53 was upregulated in the thioridazine-treated group compared with the controls (Fig. 2B). Consistent with this, the expression of anti-apoptotic, oncogenic, and anti-proliferation markers, such as Bcl-2, survivin, c-Myc, cyclooxygenase (COX-2), intercellular adhesion molecule 1 (ICAM-1), and X-linked inhibitor of apoptosis protein (XIAP), was downregulated in thioridazine-treated tumors (Fig. 2B,). Taken together, these data suggest that thioridazine plays an important role in preventing ovarian tumor progression.

### Thioridazine suppresses tumor angiogenesis *in vivo*

**Figure 3:**
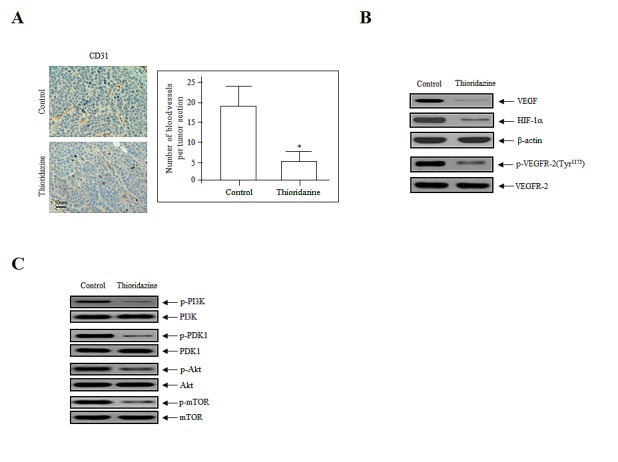
Thioridazine inhibits *in vivo* angiogenesis (A) Endothelial cells in paraffin-embedded tumor sections were stained using anti-CD31 antibodies. Thioridazine-treated tumors exhibited ~fourfold reduced CD31 staining. Bar = 50 μm. *, *P*<0.05 vs. control. (B) Thioridazine downregulated the expression of VEGF and HIF-1α and the phosphorylation of VEGFR-2, as assessed by the immunoblotting of tumor samples. β-actin was used as the loading control. (C) Thioridazine decreased the phosphorylation of major components of the PI3K/mTOR signaling pathway. Total cell lysates were prepared and analyzed by immunoblotting using anti-phospho-PI3K, -phospho-PDK1, -phospho-Akt, and -phospho-mTOR antibodies. The total non-phosphorylated proteins (PI3K, PDK1, Akt, and mTOR) were used as loading controls. Protein expression levels were analyzed by densitometry.

We reported previously that thioridazine inhibits the expression of angiogenesis-related factors *in vitro* [7]. To confirm the anti-angiogenic effects of thioridazine on tumor angiogenesis *in vivo*, the expression of proteins involved in angiogenesis was assessed in tumor cell lysates using immunohistochemistry and immunoblotting. Immunohistochemical staining of endothelial cells in tumor sections of thioridazine-treated mice revealed a ~fourfold reduction in the number of blood vessels stained with CD31 (Fig. 3A). Consistent with this, immunoblotting revealed that the expression of VEGF and hypoxia-inducible factor 1α (HIF-1α) and the phosphorylation of VEGFR-2 were reduced in thioridazine-treated tumors compared with the controls (Fig. 3B). We next analyzed the activation of downstream targets of PI3K after treatment with thioridazine using immunoblotting. Treatment with thioridazine downregulated the phosphorylation, but not total levels, of PDK1, Akt, and mTOR (Fig. 3C). These results suggest that thioridazine stimulates changes in apoptotic cell death, cell cycle progression, and the PI3K/Akt signaling pathway. Taken together, these data demonstrate that thioridazine potently inhibits angiogenesis and tumor growth *in vivo*.

## DISCUSSION

Thioridazine is used extensively to treat psychotic diseases such as psychosis and schizophrenia owing to its potent anti-anxiety and anti-psychotic effects. Recently, we demonstrated that thioridazine dramatically suppressed cell growth by inducing apoptosis, and that its angiostatic effects were mediated by the inhibition of FAK/mTOR signaling in ovarian cancer cells *in vitro* [6, 7]. In the current study, we explored the direct effects of thioridazine on anti-tumor and anti-angiogenic activity that could target the VEGFR-2/PI3K/mTOR signal transduction in ovarian tumor xenografts *in vivo.* As expected, the volume of thioridazine-treated tumors was 70% less than those of the controls. The expression of the proliferative markers PCNA and Ki-67 was significantly lower in thioridazine-treated tumors, whereas the expression of anti-apoptotic, oncogenic, and anti-proliferative proteins (including Bcl-2, survivin, c-Myc, COX-2, ICAM-1, and XIAP) was decreased significantly compared with the controls. Collectively, these results suggest that thioridazine inhibits ovarian tumor progression.

VEGF plays a role in tumor angiogenesis by activating the proliferation and migration of endothelial cells during microvessel formation in organ development [9]. In cancer, the activity of endothelial cells plays a pivotal role in regulating various vascular biological and pathological functions. Although VEGFR-1 and VEGFR-2 are structurally similar, they have distinct functions during angiogenesis. VEGFR-2 plays a vital role in activating the major downstream components responsible for cell growth, endothelial cell invasion, migration, differentiation, and embryonic angiogenesis [20-22]. In contrast, VEGFR-1 has no role in the proliferation and migration of endothelial cells [23]. In addition, HIF proteins regulate the expression of VEGF, whereas hypoxic conditions upregulate HIF-1α expression. Activated HIF-1α promotes the proliferation and invasion of endothelial cells, as well as migration and capillary tubule formation in malignant tumors. As expected, the current study revealed that VEGF and HIF-1α levels and VEGFR-2 phosphorylation were inhibited significantly in thioridazine-treated tumors compared with the controls *in vivo.* PI3K/Akt signaling plays a vital role in the various physiological functions of malignant tumors. Akt activity is modulated by PI3K, which anchors Akt to the cell membrane and allows it to be activated by PDK1 [24]. Thioridazine treatment *in vivo* downregulated the phosphorylation, but not expression, of PDK1, Akt, and mTOR.

In conclusion, the anti-tumor and anti-angiogenic effects of thioridazine were confirmed *in vivo* using mouse ovarian tumor xenografts, followed by immunoblotting and immunohistochemistry. These data supply evidence for the molecular mechanism by which thioridazine inhibits human ovarian carcinoma growth *in vivo*. Results revealed that the anti-tumor and angiostatic effects of thioridazine were regulated by reducing the phosphorylation of VEGFR-2 and inhibiting PI3K/mTOR signaling. Collectively, these results strongly suggest that thioridazine might be a novel anti-cancer and anti-angiogenic agent for ovarian carcinoma.

## MATERIALS AND METHODS

### Cell culture, mice, reagents and antibodies

Human ovarian cancer cells (2774) were obtained from the American Type Culture Collection (ATCC, Manassas, VA), and were grown in Dulbecco’s modified Eagle’s medium (DMEM; Life Technologies, Gaithersburg, MD) supplemented with 10% fetal bovine serum (FBS) and 100 U/ml penicillin/streptomycin at 37°C in a humidified 5% CO_2_ incubator. Specific pathogen-free BALB/c-nu/nu mice (5–6 weeks old) were supplied by Orientbio (Sungnam, Korea). The Institutional Animal Care and Use Committee (IACUC) at the Research Institute of the National Cancer Center approved all animal studies. Thioridazine was purchased from Sigma-Aldrich (St. Louis, MO). The following primary antibodies against the following proteins were used for western blotting: anti-caspase-3, anti-caspase-9, anti-c-Myc, anti-Bcl-2, anti-Bax, anti-p53, anti-survivin, anti-COX-2 (all from Cell Signaling Technologies, Beverly, MA), anti-PARP, anti-XIAP (both from BD Biosciences, San Jose, CA), anti-cyclin D1, anti-CDK4, anti-p16, anti-p27, anti-phospho-PI3K, anti-PI3K, anti-phospho-Akt, anti-Akt, anti-phospho-PDK1, anti-PDK1, anti-phospho-mTOR, anti-mTOR, anti-ICAM-1, anti-HIF-1α, anti-VEGF, anti-VEGFR-2, anti-phospho-VEGFR-2 (all from Santa Cruz Biotechnology, Santa Cruz, CA), and anti-β-actin (Sigma-Aldrich). In addition, the following primary antibodies were used for immunohistochemical analysis: anti-CD31 (PECAM-1), anti-Ki67, anti-cleaved caspase-3 (all from Abcam, Cambridge, UK), and anti-PCNA (Dako, Denmark).

### Mouse xenografts and immunohistochemistry

Briefly, 5–6-week-old BALB/c-nu/nu mice were implanted subcutaneously (s.c.) with 1.9 × 10^6^ 2774 tumor cells. When the tumors reached a volume of ~100 mm^3^, on day 14, animals were treated orally with 25 mg/kg thioridazine every 3 days for 27 days. Tumors were measured in three dimensions using calipers, and tumor volume (in mm^3^) was calculated using the following formula: tumor volume (mm^3^) = (*a* × *b* ^2^)/2, where *a* = length in mm, and *b* = width in mm. Body weight was measured every other day. Mice were sacrificed 1 day after the final injection. Tumors were then excised and weighed; half of each tumor was frozen, and half was fixed in 10% neutral-buffered formalin, embedded in paraffin, and sectioned for H&E staining and immunohistochemistry. The antibodies described above were used in an automatic immunohistochemical-staining instrument (Ventana, Tucson, AZ) following the manufacturer’s instructions. Sections were subsequently visualized at 200× magnification; staining was assessed in >200 cells from each section.

### Immunoblotting analysis

Tissues were collected, rinsed in PBS, and centrifuged. The pellets were then resuspended in lysis buffer (50 mM Tris pH 7.2] 150 mM KCl, 1% Triton X-100, 2 μg/ml aprotinin, 1 mM phenylmethylsulfonyl fluoride, 1 μg/ml leupeptin, and 1 μg/ml pepstatin A) containing a protease inhibitor cocktail, and incubated for 30 min. The protein concentration was determined using a Bio-Rad protein assay kit. Cell lysates were mixed with 6× SDS loading buffer, and then were separated on 10–12% gels using SDS-PAGE. The proteins were transferred to Immobilon P membranes (Millipore Corp., Billerica, MA). After blocking, the membranes were incubated for 1 h at room temperature with the indicated primary antibodies. The blots were then rinsed three times in wash buffer, and incubated with the appropriate horseradish peroxidase-conjugated secondary antibodies. The protein bands were visualized using an enhanced chemiluminescence (ECL) detection system (Amersham Biosciences, Piscataway, NJ).

### Data analysis and statistics

Values are presented as means ± SDs. Statistical comparisons between groups were performed using Student’s *t*-tests, and a *P* value of <0.05 (*) was considered to indicate statistical significance.

## SUPPLEMENTARY MATERIALS AND METHODS



## References

[1] Cowap J, Hardy J (1998). Thioridazine in the management of cancer-related sweating. J. Pain Symptom Manage.

[2] Zhukovsky DS (2002). Fever and sweats in the patient with advanced cancer. Hematol. Oncol. Clin. North. Am.

[3] Ly KL, Chidgey J, Addington-Hall J, Hotopf M (2002). Depression in palliative care: a systematic review. Part 2 Treatment. Palliat. Med.

[4] Kamiwatari M, Nagata Y, Kikuchi H, Yoshimura A, Sumizawa T, Shudo N, Sakoda R, Seto K, Akiyama S (1989). Correlation between reversing of multidrug resistance and inhibiting of [3H]azidopine photolabeling of P-glycoprotein by newly synthesized dihydropyridine analogues in a human cell line. Cancer Res.

[5] Pantazaki AA, Lialiaris TS (1999). A combined biochemical and cytogenetic study of thioridazine-induced damage to nucleic acids. Mutagenesis.

[6] Rho SB, Kim BR, Kang S (2011). A gene signature-based approach identifies thioridazine as an inhibitor of phosphatidylinositol-3’-kinase (PI3K)/AKT pathway in ovarian cancer cells. Gynecol. Oncol.

[7] Byun HY, Lee JH, Kim BR, Kang S, Dong SM, Park MS, Lee SH, Park SH, Rho SB (2012). Anti-angiogenic effects of thioridazine involving the FAK-mTOR pathway. Microvasc. Res.

[8] Folkman J, Shing Y (1992). Angiogenesis. J. Biol. Chem.

[9] Risau W (1997). Mechanisms of angiogenesis. Nature.

[10] Ferrara N (2002). VEGF and the quest for tumour angiogenesis factors. Nat. Rev. Cancer.

[11] Yoshiji H, Gomez DE, Shibuya M, Thorgeirsson UP (1996). Expression of vascular endothelial growth factor, its receptor, and other angiogenic factors in human breast cancer. Cancer Res.

[12] Xia C, Meng Q, Cao Z, Shi X, Jiang BH (2006). Regulation of angiogenesis and tumor growth by p110 alpha and AKT1 via VEGF expression. J. Cell. Physiol.

[13] Arbiser JL, Kau T, Konar M, Narra K, Ramchandran R, Summers SA, Vlahos CJ, Ye K, Perry BN, Matter W, Fischl A, Cook J, Silver PA, Bain J, Cohen P, Whitmire D, Furness S, Govindarajan B, Bowen JP (2007). Solenopsin, the alkaloidal component of the fire ant (Solenopsis invicta), is a naturally occurring inhibitor of phosphatidylinositol-3-kinase signaling and angiogenesis. Blood.

[14] Jiang BH, Liu LZ (2008). PI3K/PTEN signaling in tumorigenesis and angiogenesis. Biochim. Biophys. Acta.

[15] Duronio V, Scheid MP, Ettinger S (1998). Downstream signalling events regulated by phosphatidylinositol 3-kinase activity. Cell. Signal.

[16] Jiang BH, Zheng JZ, Aoki M, Vogt PK (2000). Phosphatidylinositol 3-kinase signaling mediates angiogenesis and expression of vascular endothelial growth factor in endothelial cells.

[17] Fingar DC, Salama S, Tsou C, Harlow E, Blenis J (2002). Mammalian cell size is controlled by mTOR and its downstream targets S6K1 and 4EBP1/eIF4E. Genes Dev.

[18] Wan X, Harkavy B, Shen N, Grohar P, Helman LJ (2007). Rapamycin induces feedback activation of Akt signaling through an IGF-1R-dependent mechanism. Oncogene.

[19] Pommier Y, Sordet O, Antony S, Hayward RL, Kohn KW (2004). Apoptosis defects and chemotherapy resistance: molecular interaction maps and networks. Oncogene.

[20] Breier G (2000). Endothelial receptor tyrosine kinases involved in blood vessel development and tumor angiogenesis. Adv. Exp. Med. Biol.

[21] Ferrara N (2002). Rloe of vascular endothelial growth factor in regulation of physiological angiogenesis. Am. J. Physiol. Cell Physiol.

[22] Meyer RD, Rahimi N (2003). Comparative structure-function analysis of VEGF-1 and VEGF-2. Ann. N. Y. Acad. Sci.

[23] Meyer RD, Singh A, Majnoun F, Latz C, Lashkari K, Rahimi N (2004). Substitution of C-terminal of VEGFR-2 with VEGFR-1 promotes VEGFR-1 activation and endothelial cell proliferation. Oncogene.

[24] Duronio V, Scheid MP, Ettinger S (1998). Downstream signalling events regulated by phosphatidylinositol 3-kinase activity. Cell. Signal.

