# Supplementary material for: Thioridazine inhibits angiogenesis and tumor growth by targeting the VEGFR-2/PI3K/mTOR pathway in ovarian cancer xenografts
Source: Oncotarget. 2014 Jun 6;5(13):4929–34. doi: 10.18632/oncotarget.2063 (PMC4148111; doi:10.18632/oncotarget.2063)
Supplement: Supplementary file 1 [file oncotarget-05-4929-s001.pdf]

Thioridazine inhibits angiogenesis and tumor growth by targeting the VEGFR-2/PI3K/mTOR pathway in ovarian cancer xenografts

MATERIALS AND METHODS

Luciferase reporter-gene assay

In vitro luciferase activity was performed as reported previously [1]. In brief, cells at 80~85% confluency were co-transfected with promoter luciferase plasmids with the *Renilla* luciferase reporter (Promega, Fitchburg, WI) for 24 h. After lysis with RIPA buffer, lysates were cleared by centrifugation for 15 min at 14,000 rpm and the cell extracts were incubated with the luciferase substrate reagent for 30 min at room temperature for 30 min according to the provided protocol. Then, a 5 μl aliquot of each sample was quantitated using MicroLumat Plus LB96V luminometer. The ratio was normalized for the *Renilla* luciferase activity in order to correct for variations in transfection efficiency. The results were assessed by three independent experiments.

Silencing of gene expression by small interference RNA

Small interfering RNA (siRNA) oligonucleotide that target vascular endothelial growth factor receptor 2 (VEGFR-2) (ON-TARGET *plus* SMARTpool) and a non-targeting siRNA pool were obtained from Millipore (Billerica, MA) and resuspended according to the manufacturer's instructions. For transfection, 8.5×10^5 HUVECs were

transfected with the oligonucleotides at a final concentration of 100 nM using Lipofectamine^{TM} 2000 Reagent (Invitrogen, Carlsbad, CA) according to the manufacturer's protocol.

REFERENCES

1. Rho SB, Song YJ, Lim MC, Lee SH, Kim BR, Park SY. Programmed cell death 6 (PDCD6) inhibits angiogenesis through PI3K/mTOR/p70S6K pathway by interacting of VEGFR-2. Cell. Signal. 2012; 24: 131-139.

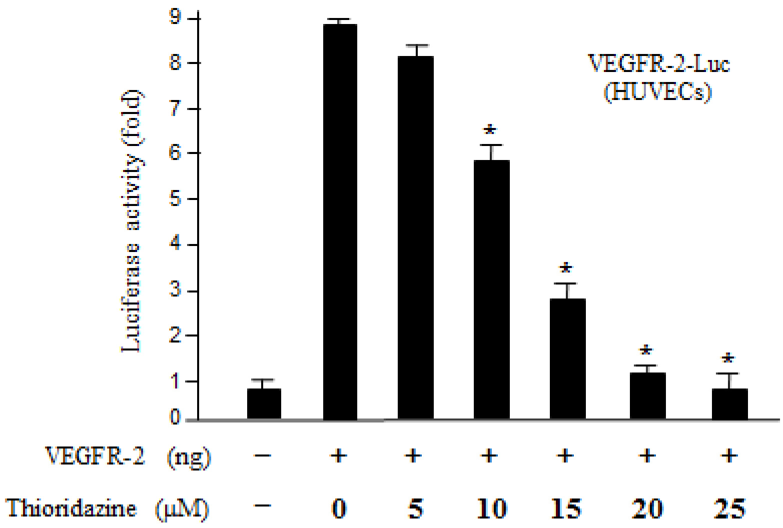

Fig S1: Inhibition of VEGFR-2-dependent transcription by thioridazine. HUVECs were co-transfected with 500 ng of VEGFR-2-Luc, 500 ng of a VEGFR-2 expression plasmid (pcDNA3.1/VEGFR-2), and increasing concentrations of thioridazine (0, 5, 10, 15, 20, and 25 μM).

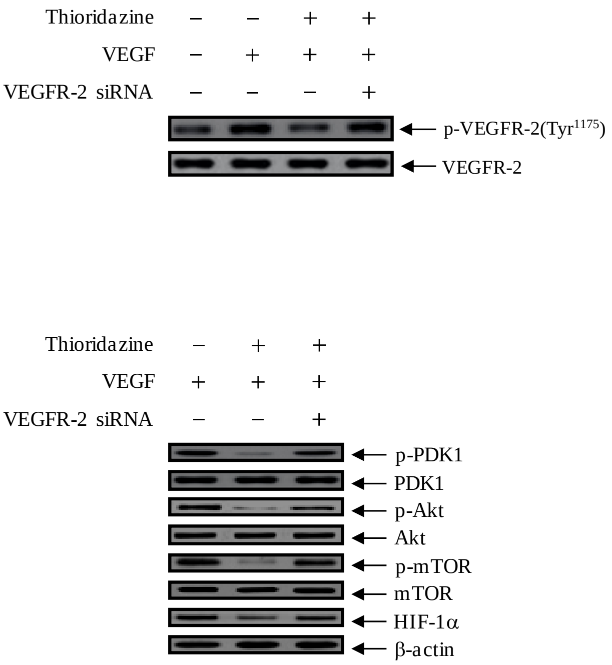

Fig S2: Thioridazine suppresses VEGF-induced VEGFR-2 and down-stream components expression of PI3K. HUVECs transiently transfected with VEGFR-2 siRNA were incubated with or without 10 ng/ml VEGF. Cells lysates were harvested and analyzed with the indicated antibodies by immunoblotting analysis, respectively (upper panel). Knockdown of VEGFR-2 recovers VEGF-induced down-stream modulators protein expression reduced by thioridazine. HUVECs transiently transfected with/without VEGFR-2 siRNA, and were seeded in the presence of 10 ng/ml VEGF. Phosphorylated PDK1, Akt, mTOR, and HIF-1α were developed by Western blot analysis (lower panel). Three independent experiments were conducted in triplicate.
